# Injectable Hydrogel as a Unique Platform for Antitumor Therapy Targeting Immunosuppressive Tumor Microenvironment

**DOI:** 10.3389/fimmu.2021.832942

**Published:** 2022-01-17

**Authors:** Yushuai Liu, Yuanyuan Geng, Beilei Yue, Pui-Chi Lo, Jing Huang, Honglin Jin

**Affiliations:** ^1^ Department of Ophthalmology, Union Hospital, Tongji Medical College, Huazhong University of Science and Technology, Wuhan, China; ^2^ College of Biomedicine and Health and College of Life Science and Technology, Huazhong Agricultural University, Wuhan, China; ^3^ Department of Biomedical Sciences, City University of Hong Kong, Kowloon, Hong Kong, SAR China

**Keywords:** cancer immunotherapy, tumor microenvironment (TME), injectable hydrogels, immunogenic cell death, abscopal effect, controlled drug release

## Abstract

Cancer immunotherapy can boost the immune response of patients to eliminate tumor cells and suppress tumor metastasis and recurrence. However, immunotherapy resistance and the occurrence of severe immune-related adverse effects are clinical challenges that remain to be addressed. The tumor microenvironment plays a crucial role in the therapeutic efficacy of cancer immunotherapy. Injectable hydrogels have emerged as powerful drug delivery platforms offering good biocompatibility and biodegradability, minimal invasion, convenient synthesis, versatility, high drug-loading capacity, controlled drug release, and low toxicity. In this review, we summarize the application of injectable hydrogels as a unique platform for targeting the immunosuppressive tumor microenvironment.

## Introduction

Cancer is a major threat to human health worldwide (1). Cancer immunotherapy has emerged as a promising cancer treatment approach that can inhibit tumor metastasis and recurrence by boosting antitumor immune responses (2, 3). Cancer immunotherapies have revolutionized the treatment of many cancer types in clinical settings. Immunotherapeutic agents include immune checkpoint inhibitors, vaccines, immunologic adjuvants, adoptive cell transfer, and nonspecific immune-stimulating factors (e.g., cytokines) (4). Nevertheless, low T cell infiltration levels, the presence of inhibitory immune cells, and the lack of neoantigens limit response to immunotherapy. Systemic administration of conventional drugs often requires high dosages or multiple injections, which can lead to severe immune-related adverse effects and low patient compliance (5–7). Multiple immunosuppressive factors in the tumor microenvironment (TME) have been shown to affect the delivery of therapeutic agents and efficacy of T cell-based therapies, thus influencing the therapeutic efficacy of cancer immunotherapy (8–10). Therefore, modulating or reprogramming the immunosuppressive TME can enhance the efficacy of cancer immunotherapy. Many studies and clinical trials aiming to target tumor immunosuppressive microenvironment to eradicate malignant cells are ongoing (10, 11).

**Figure 1:**
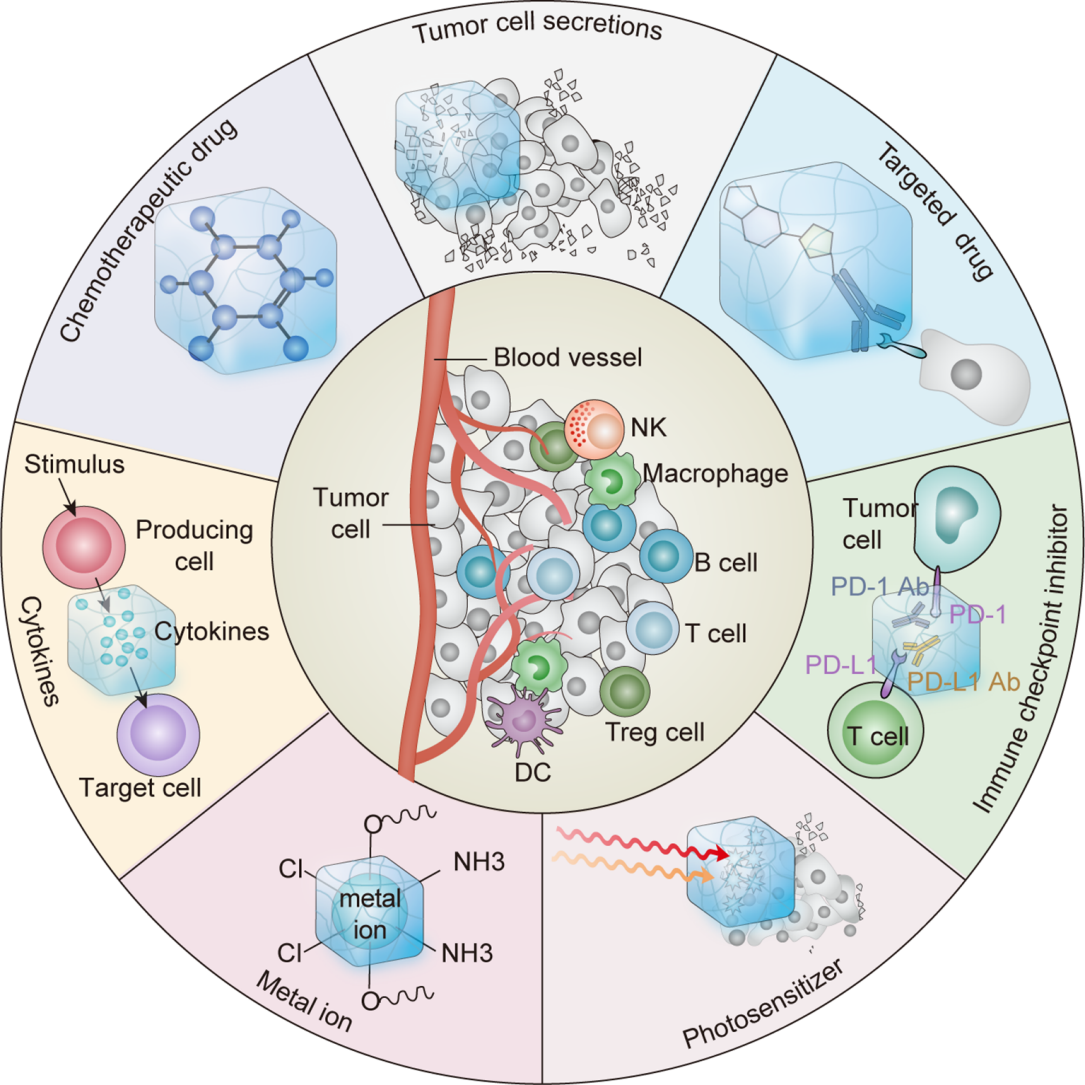
Schematic diagram of antitumor therapy platform using hydrogels as platform to elicit antitumor immune response.

Hydrogels with 3D network structures have been widely used in various fields, especially in biomedicine (7, 12–14). Injectable hydrogels have attracted considerable attention as vehicles for sustained drug delivery *in situ* because of their unique advantages, including easy delivery by syringe and minimal surgical wounds (13, 15). Injectable hydrogels can be loaded with various agents, including chemotherapeutic drugs, immunotherapeutic agents, antibodies, vaccines, cytokines, and immune cells (7, 14, 16). Sustained and controlled release of these therapeutic agents by injectable hydrogels can activate systemic antitumor immune responses and inhibit tumor metastasis and recurrence while causing minimal toxicity (7). Herein, we highlight recent advances in reprogramming the immunosuppressive TME using injectable hydrogels to improve the efficacy of cancer immunotherapy (**Figure 1**).

## Categories of Injectable Hydrogels

Injectable hydrogels are usually formed by quick sol-gel phase transition or chemical polymerization *in situ*. They can be directly delivered into the target sites by injection (12, 16). Injectable hydrogels can be classified into chemically and physically cross-linked hydrogels based on the gelling mechanism (13, 16). Chemically cross-linked injectable hydrogels are generated by introducing covalent linkages between polymer chains *via* disulfide formation, photo-irradiation, enzymes, Schiff’s base reactions, Michael-type addition reactions, or Diels-Alder reactions (16). On the other hand, physically cross-linked injectable hydrogels are formed through intermolecular interactions, such as hydrogen bonds, hydrophobic interactions, ionic cross-linking, and host-guest interaction (16). Injectable hydrogels can also be classified as natural or synthetic hydrogels based on the polymers used for their preparation (7). Natural injectable hydrogels are typically composed of polysaccharides, proteins, and DNA. In contrast, synthetic hydrogels consist of biodegradable polymers, such as polypeptides and polyesters (7). Additionally, injectable hydrogels can be divided into ordinary hydrogels and smart hydrogels according to their responses to external stimuli. Ordinary injectable hydrogels are not sensitive to environmental changes, whereas smart injectable hydrogels can be affected by temperature, pH, enzyme, and photoelectricity (13, 17). Moreover, injectable hydrogels can be biologically functionalized with targeting moieties that have an affinity for unique or overexpressed tumor cell markers for targeted drug delivery applications (18).

Over the past decade, many studies have investigated the antitumor potential of drug-loaded hydrogels (19). The therapeutic potential of hydrogels has also been investigated in patients with cancer. Up to September 2021, four clinical studies related to drug-loaded hydrogels for the treatment of cancer have been registered in the US registry of clinical trials (https://clinicaltrials.gov/). Two completed, open-label, dose escalation clinical studies (NCT02891460, NCT02307487) evaluated the efficacy of mitomycin C-loaded hydrogels (TC-3) in patients with bladder cancer. The results of these studies have not been published yet (**Table 1**).

**Table 1:** Drugs embedded in hydrogels were used to treat cancers based clinical trials up to September 2021.

Study title	Conditions	Status	Identifier
A Prospective Open Label Comparative Dose Ranging Study Evaluating the Effect of Pre-TURBT Intravesical Instillation of Mitomycin C (MMC) Mixed with TC-3 Gel in Patients with Non Muscle Invasive Bladder Cancer (NMIBC)	Bladder Cancer	Withdrawn	NCT01799499
Safety and Tolerability Study Which Evaluate Intravesical Instillation with Mitomycin C Mixed with TC-3 Drug Retaining Hydrogel Device in Patients with Muscle Invasive Bladder Cancer	Bladder Cancer	Completed	NCT02891460
Safety of Pre-TURBT Intravesical Instillation of Escalating Doses of TC-3 Gel and MMC in NMIBC Patients	Bladder Cancer	Completed	NCT02307487
Safety and Efficacy of Doxorubicin-eluting-bead Embolization in Patients with Advanced Hepatocellular Carcinoma	Hepatocellular Carcinoma	Unknown	NCT02525380

## Immunosuppressive Status of TME

TME is an integral part of tumors and can affect the efficacy of cancer treatment (9). At different stages of tumor development, different immune cell types are present in the TME. At an early stage, tumors are infiltrated by antitumor immune cells, including macrophages, natural killer (NK) cells, lymphocytes, and dendritic cells (DCs) (20). However, at later stages of tumor development, antitumor immune responses are hindered by immunosuppressive cells, such as myeloid-derived suppressor cells (MDSCs), regulatory T cells (Tregs), and M2 macrophages (20, 21). The balance between different types of immune cells determines the outcome of antitumor immune responses.

CD8^+^ cytotoxic T lymphocytes (CTLs) and CD4^+^ T helper (Th) cells are paramount immune cells for tumor cell elimination (22). Th1 responses, characterized by the production of IFN-γ, TNF-α, and IL-2, are also essential for tumor rejection. However, Th1 responses can also contribute to tumor escape *via* IFN-γ-induced expression of the checkpoint molecule programmed death-ligand 1 (PD-L1) or tumor immunoediting and selection of resistant clones (23). In addition, long-term exposure of tumor antigens to Th1 cells and other T cell subtypes may promote the expression of inhibitory receptors, such as PD-L1, lymphocyte activation gene 3 protein (LAG-3), and T-cell immunoglobulin (Ig) domain and mucin domain protein 3 (TIM-3) (24). Immune checkpoint pathways in cancer cells can cause T-cell dysfunction and immune evasion. Immune checkpoint blockade (ICB), especially antibodies against cytotoxic T-lymphocyte-associated protein 4 (CTLA-4), programmed cell death protein 1 (PD-1), and PD-L1, can reverse immunosuppression and prevent immune evasion (9). ICB has shown remarkable long-term survival benefits in cancer patients with several types of tumors, including melanoma, non-small cell lung cancer, and renal cell carcinoma (16, 25).

However, Tregs, another subset of CD4^+^ T cells, often inhibit antitumor immune responses and promote tumor growth. Tregs can directly interact with CTLs and NK cells or indirectly inhibit the antitumor activity of CTLs and NK cells by producing immunoregulatory cytokines, such as IL-10 and TGF-β (10). Notably, Tregs have been associated with unfavorable survival in patients with many types of cancer (26). Hence, eliminating Tregs in the TME may enhance antitumor immune responses. Th2 cells can also block T-cell-induced tumor rejection by promoting T-cell anergy, suppressing T-cell-mediated cytotoxicity, and enhancing humoral immunity (10).

Tumor cells promote the recruitment of bone marrow-derived cells (BMDCs), which can differentiate into tumorigenic cell subtypes under certain conditions (20). For instance, tumor-associated macrophages (TAMs) derived from BMDCs promote tumor progression by facilitating angiogenesis, invasion, and metastasis *in vivo* (27). MDSCs, another type of BMDCs, can suppress antitumor immune responses by inhibiting T cells and NK cells and promoting the expansion of Treg populations within the TME (21).

## Injectable Hydrogels Targeting Immunosuppressive Tumor Microenvironment

### Targeting Immune Checkpoint Molecules

Immune checkpoint blockade (ICB) immunotherapies, especially antibodies against CTLA-4, PD-1, and PD-L1, have revolutionized cancer treatment (28). However, ICB monotherapies show limited efficacy in most patients and may cause significant toxicity (6, 9, 29). Therefore, more effective and safer combination therapies involving ICB are under development. PD-L1 expressed on the surface of tumor cells and on antigen-presenting cells can interact with PD-1 expressed on activated T cells, promoting T-cell apoptosis, anergy, and exhaustion (30, 31). Blocking the PD-1/PD-L1 pathway with anti-PD-1 or anti-PD-L1 antibodies has demonstrated promising therapeutic efficacy in a variety of tumor types (32–35); however, response rates are only 10%–30% (29, 36). Low neoantigen burden, insufficient infiltration of tumor-specific T cells, and low expression of PD-L1 may contribute to the low response rates in cancer patients treated with ICB (20, 37–41). Moreover, multiple administration cycles of anti-PD-1 antibodies can induce severe immune-related side effects (42–44); local delivery of antibodies can minimize off-target effects and increase drug bioavailability (45).

**Figure 2:**
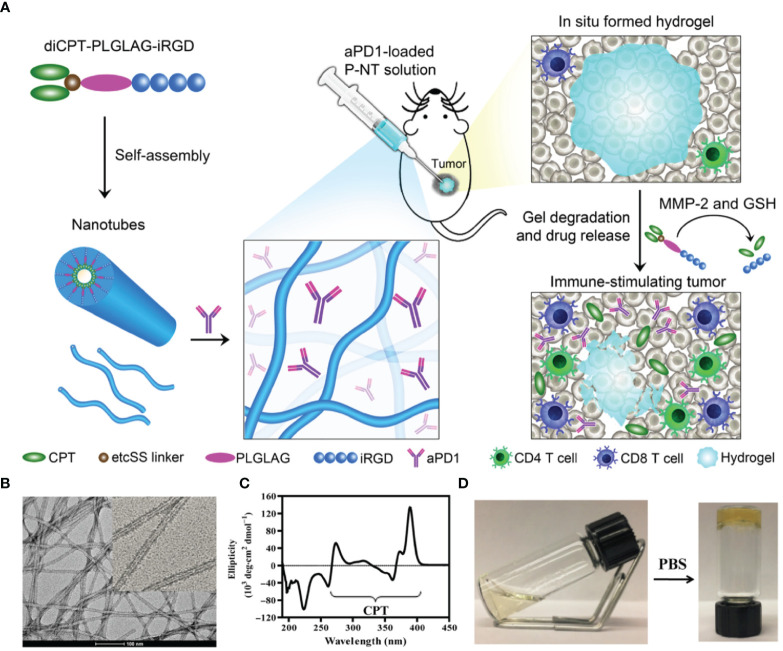
Schematic illustration of the *in situ* formed P-NT-anti-PD-1 hydrogel. **(A)** *In situ* formation of P-NT-anti-PD-1 hydrogel, which enables localized CPT and anti-PD-1 delivery and promotes the activation of CD4 and CD8 T cells in the tumor microenvironment. **(B)** Representative transmission electron microscopy (TEM) image of the networks of the P-NT hydrogel. **(C)** Circular dichroism (CD) spectrum of camptothecin (CPT) solution. **(D)** Photographs of liquid P-NT transformed into hydrogel after the addition of phosphate-buffered saline (PBS). Reprinted with permission from Science Advances (46).

Wang et al. developed a drug-based supramolecular hydrogel for local delivery of immune checkpoint inhibitors (ICIs) to boost the host’s immune system against tumors (**Figure 2**) (46). They first synthesized the amphiphilic prodrug, diCPT-PLGLAG-iRGD, by conjugating a hydrophilic iRGD. This prodrug can spontaneously assemble into supramolecular nanotubes (P-NTs). By mixing a therapeutic dose of anti-PD-1 antibodies and P-NTs, they developed a hydrogel loaded with anti-PD-1 antibodies. Wang et al. found that this formulation could serve as a reservoir for long-term release of camptothecin (CPT) and anti-PD-1 antibodies within the TME, thereby inducing a potent antitumor immune response. They also found that local P-NT-anti-PD-1 treatment in GL-261 brain cancer and CT 26 colon cancer models led to tumor regression in 100% of mice.

The low immunogenicity of some tumor types and the body’s decreased immune responses to tumor limit the development of immunotherapy. Immunogenic cell death (ICD), featured by the release of tumor-associated and tumor-specific antigens, danger-associated molecular patterns, and pro-inflammatory cytokines, plays an essential role in cancer immunotherapy (47). Recent evidence suggests that neoadjuvant chemotherapy and the use of biomaterials-based delivery systems both enhance the therapeutic efficacy of immunotherapy owing to the induction of ICD (48, 49). Gu et al. engineered an injectable reactive oxygen species (ROS)-responsive hydrogel co-loaded with gemcitabine (GEM) and anti-PD-L1 antibodies for *in situ* chemo-immunotherapy (50). As the scaffold consists of ROS-degradable hydrogel and the TME contains high levels of ROS, GEM and anti-PD-L1 antibodies can be specifically released in the TME. In B16-F10 melanoma and 4T1 breast tumor (low-immunogenic) mouse models, local GEM delivery increased tumor immunogenicity and augmented the antitumor efficacy of ICB, thereby promoting tumor regression and suppressing tumor recurrence. To enhance the expression of tumor-associated antigens, Ruan et al. developed an *in situ* formed dual-bioresponsive gel depot for co-delivery of anti-PD-1 antibodies and zebularine (Zeb), a demethylation agent that enhances the expression of tumor-associated antigens (51). Anti-PD-1 antibodies were loaded into pH-sensitive CaCO_3_ nanoparticles (anti-PD1-NPs) and encapsulated with Zeb in the ROS-responsive hydrogel (Zeb-anti-PD-1-NPs-Gel). Local release of Zeb increased the immunogenicity of cancer cells and decreased immunosuppression. By doing so, Zeb boosted the ability of anti-PD-1 antibodies to induce T cell-mediated antitumor immune responses, inhibiting tumor growth and prolonging survival in mice bearing B16-F10 tumors. In addition to direct use of anti-PD-1 antibodies to block the PD-1/PD-L1 pathway, targeting of a specific pathway that involves PD-L1 transcriptional repressors is also practicable. Li et al. reported a cancer cell membrane-derived hydrogel scaffold loaded with Ca^2+^ channel inhibitor dimethyl amiloride (DMA) and cyclin-dependent kinase 5 inhibitor roscovitine for cancer treatment. In this system, cancer cell membrane, DMA and roscovitine were chosen with the aim of creating an antigen depot, suppressing Ca^2+^-governed exosome secretion and down-regulating tumor cell PD-L1 expression, respectively (52).

CTLA-4 is expressed on activated Th1 cells and CTLs, and binds to co-stimulatory molecules CD80 and CD86 of antigen-presenting cells, thereby inhibiting the activation and proliferation of T cells (53). Although blocking CTLA-4 signaling unleashes antitumor immune responses, systemic administration of anti-CTLA-4 antibodies may cause severe immune-related adverse events (5, 54–57). Chung et al. evaluated thermosensitive poloxamer 407 (P407) hydrogels as a slow-release system for optimizing anti-CTLA-4 therapy (58). They found that P407 hydrogel-mediated delivery of anti-CTLA-4 antibodies reduced serum antibody levels, mitigated the side effects of ICB, and exerted antitumor effects in mice bearing CT26 tumors. Similarly, Harui et al. found that local administration of hydrogel-encapsulated anti-CTLA-4 antibodies exhibited enhanced efficacy and minimal systemic toxicity in mice with MC-38 tumors (59). Peritumoral administration of 100 µg of anti-CTLA-4 antibodies loaded in hydrogels had similar or greater effects than systemic administration of 600 µg of antibodies. While preserving antitumor activity, serum exposure following the administration of hydrogel-encapsulated anti-CTLA-4 was only 1/16th of that following systemic therapy.

Song et al. developed an injectable PEG-b-poly(L-alanine) (PEA) hydrogel to co-deliver a tumor vaccine consisting of tumor cell lysates (TCLs), granulocyte-macrophage colony-stimulating factor (GM-CSF), and anti-CTLA-4 antibodies and anti-PD-1 antibodies (60). TCLs, GM-CSF, anti-CTLA-4 antibodies, and anti-PD-1 antibodies were encapsulated into the porous PEA hydrogel by mixing these agents with PEA aqueous solution. Sustained release of tumor antigens and GM-CSF promoted the recruitment and activation of DCs *in vivo*, inducing tumor-specific CTL responses. The extended release of ICIs from the hydrogel further enhanced T-cell activation and reduced Treg levels in the TME by blocking PD-1 and CTLA-4 pathways. Notably, the hydrogel-based combination therapy exhibited greater antitumor effects than the vaccine alone or ICB monotherapy in melanoma and 4T-1 mouse models.

### Targeting Tumor-Associated Macrophages

Tumor-associated macrophages (TAMs) are a key component of the TME and play a significant role in tumor progression (61, 62). There are two main subtypes of TAMs: classically activated M1 macrophages (M1-TAMs) and alternatively activated M2 macrophages (M2-TAMs). M1-TAMs, which express high levels of IL-12 and IL-23, can scavenge foreign antigens and kill tumor cells (63). Tumor cells typically promote polarization of TAMs toward M2 in TME, facilitating IL-10 production and tumor growth (8). The balance between M1 and M2 TAMs has been associated with drug resistance, angiogenesis, and immunosuppression in tumors (8). Most macrophage-targeting therapies have three goals (9, 64): (1) inhibit macrophage recruitment by blocking the C-C motif chemokine ligand 2 (CCL2)/C-C motif chemokine receptor 2 (CCR2) axis (65, 66); (2) deplete macrophages or block -factor (CSF)-1/CSF-1R signaling (67, 68); (3) reprogram TAMs toward an M1-like phenotype using melittin (69), IFN-γ (70), CD40 agonists (71), or tumor hypoxia-targeting agents (72). As macrophages are present throughout the body, systemic modulation of macrophages can lead to off-target effects and systemic toxicity (73). Furthermore, CCL2/CCR2- and CSF-1/CSF-1R-targeting strategies often result in the development of monocyte and macrophage populations that enhance neoangiogenesis and metastasis (74, 75).

M2-TAM depletion has proved effective in promoting tumor regression by suppressing TAM-associated immunosuppression (8). Although melittin is a potent anticancer agent, its hemolytic effects limit its clinical application. To overcome this obstacle, we developed a melittin-RADA32 hybrid peptide hydrogel. The melittin- and doxorubicin (DOX)-loaded peptide hydrogel (melittin-RADA32-DOX, or MRD hydrogel) exerted potent anti-melanoma effects by modulating the TME (76). Moreover, MRD hydrogels loaded with melittin and DOX exhibited direct cytotoxic effects, specifically depleted M2-like macrophages, and induced robust and long-lasting innate and adaptive immune responses. Notably, a single injection of the formulation significantly reduced the growth of primary melanoma tumors.

**Figure 3:**
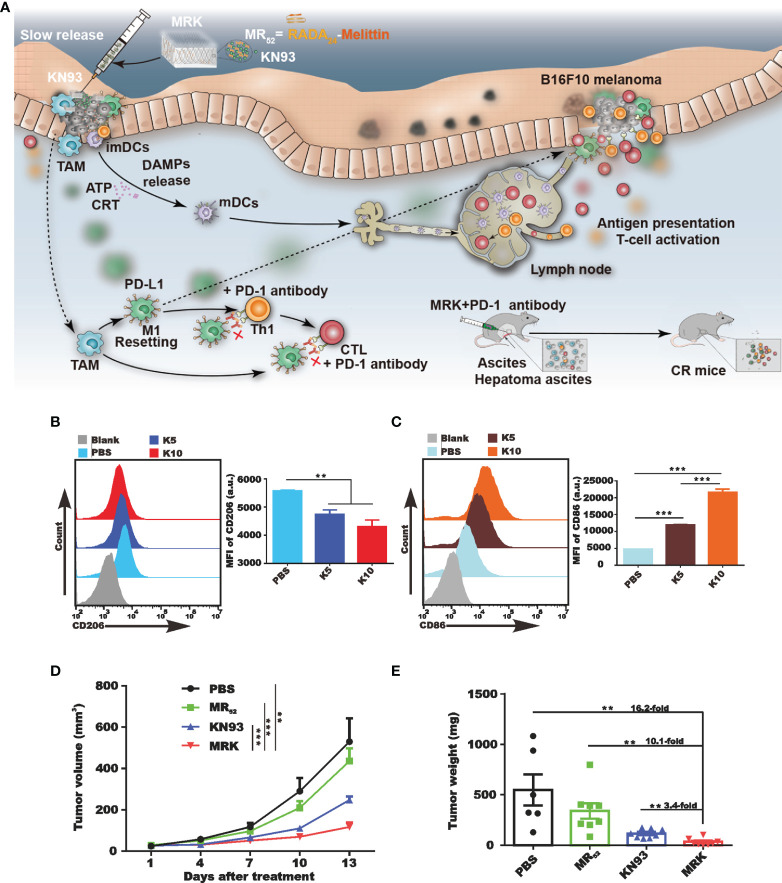
*In vivo* activation of the immune system of tumor-bearing mice by MRK. **(A)** Schematic diagram summarizing the therapeutic effects of the MRK hydrogel alone or combined with anti-PD-1 antibodies. Subcutaneous injection of MRK stimulates dendritic cell maturation and T cell activation in the lymph nodes. Activated T cells eliminate tumor cells. MRK can also stimulate M1-type polarization of tumor-associated macrophages, activating Th1 cells and cytotoxic T lymphocytes. MRK combined with PD-1 alleviates hepatocellular ascites in mice. **(B, C)** Comparison of the production of M2-type macrophages **(B)** and dendritic cells **(C)** in each group. **(D, E)** Tumor volume **(D)** and weight **(E)** in different groups. Reprinted with permission from Theranostics (78). **P < 0.01, ***P < 0.001.

External stimuli can stimulate the reprogramming of M2-TAMs into M1-TAMs, which have tumoricidal effects (77). KN93, a specific inhibitor of CAMKII, was found to have a direct tumoricidal activity and the ability to induce macrophage reprogramming (78). To further potentiate these effects of the melittin-RADA32 hydrogel, we designed a melittin-RADA24 peptide hydrogel loaded with KN93 (MR52-KN93; MRK hydrogel) (79). Compared with free KN93, the MRK hydrogel was more potent in eliminating tumor cells and inducing immunogenic cell death. Moreover, MRK significantly reduced the portion of M2-like TAMs and increased the ratio of M1-like to M2-like TAMs in the TME (**Figure 3**).

The TME is usually acidic due to the presence of hypoxia and glycolytic metabolism (79, 80). Cancer cell-derived lactate plays a critical role in the polarization of macrophages from the M1 phenotype to the M2 phenotype, which promotes tumor growth and metastasis (80). Liao et al. found that methylcellulose hydrogels loaded with lactate oxidase promoted lactate depletion and lactate-mediated repolarization of macrophages (81).

Several recent studies reported the direct involvement of TAMs in tumor resistance to ICB. By comparing the TME of ICB-resistant and ICB-sensitive murine tumors, Muraoka et al. found that TAMs in resistant tumors lacked antigen-presenting activity (82). They also found that cholesteryl-modified pullulan nanogels could efficiently deliver large peptides to TAMs and that upon TLR stimulation, the nanogel system elicited antigen-presenting activity in TAMs (82). By modulating TAMs, this formulation transformed ICB-resistant tumors into ICB-sensitive tumors. These results strongly support targeting TAMs as a promising strategy for enhancing the efficacy of cancer immunotherapy.

Because M1-TAMs can promote tumor rejection, direct injection of M1-TAMs can significantly cause tumor regression *in vivo*; however, the induction of acute inflammatory responses limits the clinical translation of this approach (83). To improve this strategy, Guerra et al. employed a synthetic extracellular matrix (ECM) system consisting of cross-linked PEGdA and Gel-PEG-Cys as a carrier for local delivery of activated M1 macrophages. They found that M1-loaded hydrogels promoted apoptosis in hepatocellular carcinoma cells and tumor regression *in vivo* while exhibiting low immunogenicity, high biocompatibility, and improved release kinetics (84).

### Targeting the Tumor Vasculature

Normal vascularization is critical for nutrients and oxygen supply, as well as metabolic waste removal. However, abnormal vascularization characterized by immature, disorganized, and permeable blood vessels creates a hostile TME characterized by hypoxia, low pH, low interstitial fluid pressure, decreased immune cell infiltration and activity, and increased risk of metastasis (85, 86). Furthermore, abnormal vascularization reduces the diffusion of chemotherapeutic drugs and impairs the efficacy of radiotherapy (86). Therefore, vascular normalization could restore tumor perfusion and oxygenation and enhance the efficacy of chemotherapy and radiotherapy (87, 88).

Antibodies against vascular endothelial growth factor (VEGF) have emerged as a promising therapeutic strategy for solid tumors, as tumor growth and metastasis require neoangiogenesis (89). Targeting VEGF signaling induces tumor vasculature normalization, further reprogramming the immunosuppressive TME and increasing the number of tumor-infiltrating lymphocytes (TILs) (90, 91). Bevacizumab, the first approved anti-VEGF drug to inhibit tumor angiogenesis in the United States, has a limited half-life and membrane permeability. To overcome these limitations, Ferreira and coworkers designed a bevacizumab-loaded alginate hydrogel for localized anti-VEGF cancer therapy by mixing alginate solution with bevacizumab and cross-linking it with calcium chloride (92). The tridimensional hydrogel increased drug stability, especially in acid environments, and provided slow and continuous drug release to the tumor and surrounding tissues after local application. Moreover, with the development of photodynamic therapy (PDT), it has shown the potential to trigger local and systemic antitumor immune responses. However, abnormal angiogenesis and hypoxia in TME promote immunosuppression. The immune response after routine PDT is usually insufficient to cause tumor regression, which limits the efficacy of PDT. Based on this, Zhou et al. developed a prolonged oxygen-generating phototherapy hydrogel (POP-Gel) system by combining the photosensitizer-loaded thermosensitive hydrogel with calcium superoxide and catalase to relieve tumor hypoxia. Long-term effective oxygen supply improved the hypoxic state of TME and down-regulated the expression of HIF-1α and VEGF, further inducing a robust antitumor adaptive immune response (93).

RNA interference (RNAi) enables robust and specific gene silencing, providing a promising therapeutic avenue for cancer treatment. However, efficient drug delivery systems for short interfering RNAs (siRNAs) are lacking (94–96). Fujii et al. developed a self-assembled nanogel of cholesterol-bearing cycloamylose with a spermine group (CH-CA-Spe) as a carrier to deliver VEGF-specific siRNAs (siVEGFs) into tumor cells. This system showed low toxicity in patients, efficient intratumor delivery, and high stability *in vivo* (97). The siVEGF-nanogel complex was taken up by tumor cells *via* the lysosomal pathway and suppressed VEGF expression in renal cell carcinoma cells. Intratumoral injections of the complex effectively suppressed tumor growth and neovascularization. The treatment also significantly suppressed MDSC infiltration and IL-17A production in the spleen, suggesting that silencing of VEGF locally in the tumor may modulate systemic immune responses.

Despite promising findings in preclinical models, the efficacy of anti-angiogenic therapies in the clinic has been disappointing, as most patients exhibit innate or acquired resistance to the treatment (98). However, anti-angiogenic therapeutics can increase the efficacy of immunotherapy (99). Additionally, low doses of anti-VEGF antibodies can induce vascular normalization, prevent the differentiation of TAMs toward an immune inhibitory M2-like phenotype, and block VEGF-mediated inhibition of DC maturation (90). Therefore, vascular normalization with anti-angiogenic therapies in combination with other therapies may be an attractive therapeutic strategy. Pal et al. developed a biocompatible self-assembled lithocholic acid dipeptide-derived hydrogel (TRI-Gel), which provided sustained delivery of DOX, anti-angiogenic combretastatin-A4 (CA4), and dexamethasone (100). TRI-Gel therapy inhibited cancer cell proliferation, angiogenesis, and inflammation at the tumor site, thereby suppressing tumor progression and prolonging median survival with reduced drug resistance (100). Yu et al. designed an *in situ* thermo-gelling hydrogel (mPEG-b-PELG) to co-deliver combretastatin A4 disodium phosphate (CA4P) and cisplatin (CDDP) for the local treatment of colon cancer (101). Compared with the free drugs, the CA4P and CDDP co-loaded gel induced less tumor cell death *in vitro*, while its antitumor effect was highest in C26 tumor-bearing mice after peritumoral injection (101).

**Figure 4:**
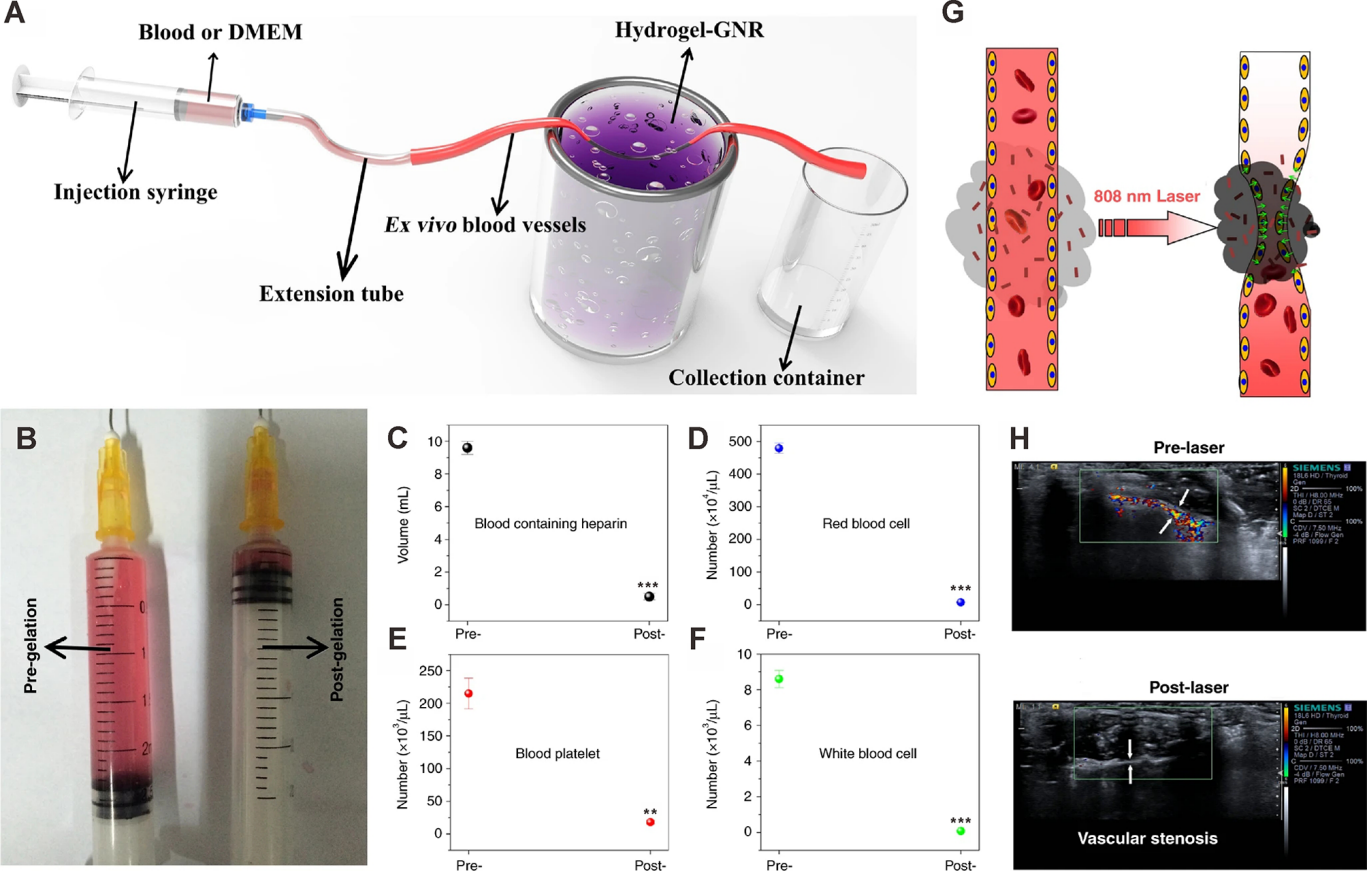
Occlusion of blood supply in ex vivo and *in vivo* artery models. **(A)** Schematic of the experimental apparatus for evaluating vessel occlusion ex vivo in blood vessels (with an inner diameter of 1.00 mm) treated with the gelation shrinkage-induced internal stress platform. **(B)** Traversed volume of DMEM in the ex vivo blood vessel model before (left) and after (right) gelation of hydrogel-GNR. **(C–F)** Collected blood volume **(C)**, red blood cells **(D)**, blood platelet **(E)**, and white blood cells **(F)** traversed through ex vivo blood vessels using rabbit blood containing heparin. **P<0.01 and ***P<0.001 compared to pre-gelation; determined using Student’s t-test. **(G)** Schematic representation of the extravascular gelation shrinkage-induced internal stress system irradiated with 808 nm laser. **(H)** CDFI images of abdominal arteries of nude mice treated which hydrogel-GNR. CDFI images were captured before and after irradiation with an 808 nm laser. White arrows indicate the blood vessels of the abdominal artery. Reprinted with permission from Springer Nature (104).

Starvation therapies can inhibit tumor progression by decreasing nutrient supply indispensable for tumor growth (102, 103). Blood vessel occlusion can permanently occlude blood and nutrition supply to the tumor. However, this strategy is often associated with poor persistence, frequent tumor metastasis and recurrence, and embolism in normal blood vessels. Zhang and coworkers established an extravascular gelation shrinkage-derived internal stress strategy to narrow blood vessels, occlude blood and nutrition supply, reduce vascular density, induce hypoxia and apoptosis, and ultimately promote starvation of the tumor (104). To this end, they engineered an organic-inorganic composite hydrogel consisting of PEG-SH-modified gold nanorods (GNR-PEG-SH) and thermal-sensitive hydrogel mixture (chitosan (CS)/mPEG-Mal/pNIPAAm-co-AAc; hydrogel-GNR). When irradiated with an 808 nm laser, hydrogel-GNR induced internal stress, which narrowed intratumor and adjoining blood vessels in a GNR-dependent manner. This starvation therapy inhibited tumor progression in both PANC-1 pancreatic cancer and 4T1 breast cancer mouse models. Importantly, this starvation strategy suppressed tumor metastasis and tumor recurrence by reducing vascular density, occluding blood and nutrition supply (**Figure 4**).

### Targeting Other Immunoregulatory Cells and Factors

In view of the strong immunosuppressive effect of Tregs in the TME, targeting Tregs has emerged as an attractive strategy to unleash antitumor immune responses and reenforce immune-mediated tumor rejection (10). Tumor-specific Tregs residing at the TME express high levels of CTLA-4 and OX40, and *in situ* injection of anti-CTLA-4 and anti-OX40 together with CpG can deplete tumor-infiltrating Tregs (104). This *in situ* immunomodulation approach activates systemic antitumor immune responses more effectively than systemic immunomodulation strategies (105). The co-delivery of tumor anti-CTLA-4, anti-PD-1, and tumor vaccines using injectable PEG-b-poly(L-alanine) hydrogels increased the efficacy of immunotherapy by reducing the number of Tregs and increasing the number of activated CD8^+^ T cells in the TME (60). In addition to directly killing tumor cells, some chemotherapeutic agents can regulate the immune system through various mechanisms, including the modulation of Tregs (106–112). Co-delivery of DOX and CpG self-crosslinking nanoparticles (CpG NPs) using injectable α-cyclodextrin/polyethylene glycol hydrogels increased the number of cytotoxic CD8^+^ T lymphocytes and decreased the numbers of MDSCs, M2-TAMs, and Tregs in the TME (107). Additionally, although chemotherapy alone reduced the number of Tregs to some extent, combination therapy using α-cyclodextrin/polyethylene glycol hydrogels-CpG NP-DOX remarkably reduced the number of Tregs in the TME (107).

**Figure 5:**
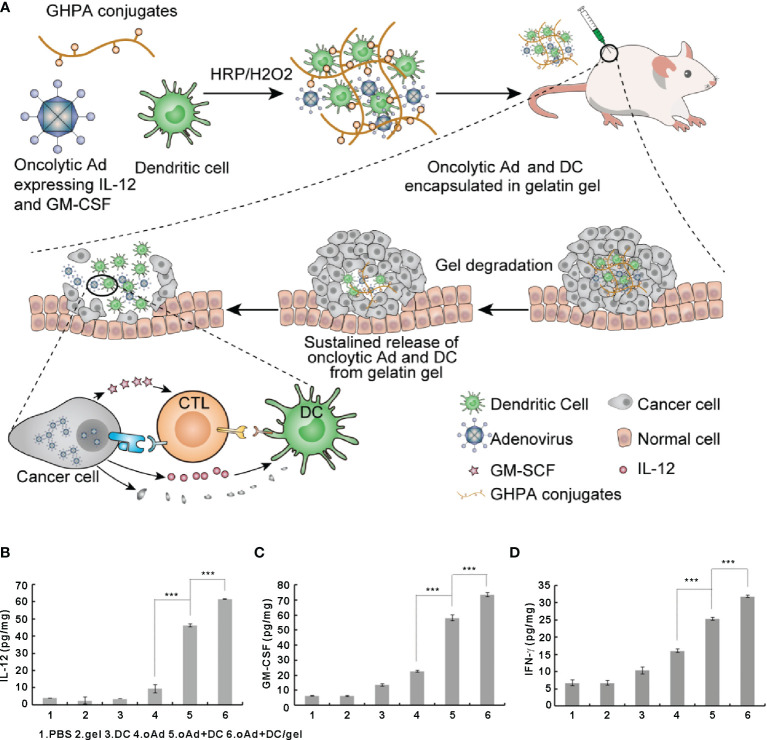
Oncolytic adenoviruses and dendritic cells encapsulated in gelatin gels activate the immune system to eliminate tumor cells and induce the expression of IL-12 and GM-CSF in tumor cells. **(A)** Schematic representation of optimized biodegradable polymeric reservoir-mediated local and sustained co-delivery of dendritic cells and oncolytic adenovirus expressing IL-12 and GM-CSF. **(B–D)** The expression levels of IL-12 **(B)**, GM-CSF **(C)**, and IFN-γ **(D)** in tumors. Reprinted with permission from Elsevier (116). ***P < 0.001.

The balance between different immune cell subsets, immune factors, and signaling molecules determine the outcome of antitumor immune response. Intratumoral delivery of immunomodulatory cytokines has been tested in the clinic as a strategy to augment antitumor immune responses (10). To elicit a therapeutic response, sufficient concentrations and long-lasting release of cytokines in TME are necessary, along with a non-toxic concentration of the cytokine outside of TME. GM-CSF, IL-2, IL-12, and IFN-γ are among the several cytokines tested for local cancer treatment based on injectable hydrogels (16). Son et al. demonstrated that GM-CSF improved the function of antigen-presenting cells and enhanced antitumor immune responses (113). Co-delivery of GM-CSF and anticancer drugs using a chitosan-based hydrogel system resulted in a synergistic anticancer effect, as tumor-specific CD8^+^ T cell responses were significantly enhanced (113). Den Otter et al. developed physically cross­linked dextran hydrogels for the local delivery of IL­2. The system exhibited a strong therapeutic effect, enhancing the clinical applicability of IL-2 (114). Kurisawa and co­workers developed an injectable hyaluronic acid­tyramine (HA­Tyr) conjugate hydrogel to locally deliver IFN-α2a to treat liver cancer (115). The enzymatically cross­linked HA­Tyr hydrogel released IFN-α2a in the TME and inhibited tumor growth while providing tunable hydrogel stiffness and rapid gelation rate (115). Eonju Oh et al. utilized gelatin-based hydrogels for sustained co-delivery of DCs and oncolytic adenovirus (oAd) co-expressing IL-12 and GM-CSF while preserving the biological activity of the cytokines (116). Compared with single treatment (oAd or DC) or combination treatment without the gel (oAd+DC), oAd+DC/gel treatment resulted in a significantly higher expression of IL-12, GM-CSF, and IFN-γ in tumors through a positive feedback loop. The high levels of IL-12, GM-CSF, and IFN-γ in the TME strongly activated endogenous and exogenous DCs, which migrated to the draining lymph nodes and promoted the activation and infiltration of CD4^+^ and CD8^+^ T cells into the tumor, finally leading to robust tumor regression. Interestingly, oAd+DC/gel treatment also alleviated tumor-induced thymic atrophy (**Figure 5**).

Chronic inflammation in TME can promote cancer progression in several ways, and remission of chronic inflammation can help control the tumor (117). The cyclooxygenase 2 (COX2) inhibitor celecoxib has been shown to exert antitumor effects in various human cancers (118, 119). For instance, simultaneous and local administration of anti-PD-1 monoclonal antibodies and celecoxib using alginate hydrogels resulted in stronger antitumor effects than anti-PD-1 or celecoxib alone. In addition, the formulation elicited a potent and sustained antitumor immune response (120). Notably, co-delivery of celecoxib and anti-PD-1 monoclonal antibodies increased the numbers of INF-γ-expressing CD4^+^ and CD8^+^ T cells and decreased the numbers of intratumoral Tregs, MDSCs, and PD-L1-positive tumor cells. Furthermore, this co-delivery system enhanced the expression of the anti-angiogenic chemokines CXCL9 and CXCL10 and suppressed the intratumoral production of IL-1, IL-6, and COX2, suggesting reduced inflammation and angiogenesis in the tumor.

## Conclusion

Numerous injectable hydrogels have been developed over the past years (121). Injectable hydrogels offer many advantages, including good biocompatibility and biodegradability, minimal invasion, convenient synthesis, versatility, high drug-loading capacity, and controlled drug release ability (122). Owing to their unique properties, injectable hydrogels can be used as drug delivery systems, which can locally and continuously release therapeutic agents. Although intratumoral injections suffer from localized treatment and inhomogenous distribution across tumors, injectable hydrogels as drug delivery systems can overcome many limitations of current systemic therapies for cancer, especially systemic toxicity and limited efficacy (123). Compared with intravenous delivery, the intratumoral injection can provide direct contact with tumor cells and immune cells, eliciting a more strong and long-lasting immune response. Besides localized treatment for single tumor, injectable hydrogels can be applied for the treatment of extensive pleural and peritoneal metastasis, such as malignant pleural effusion and malignant ascites. More importantly, in some cases, injectable hydrogels can not only effectively promote ICD of tumor cells and reshape immunosuppressive TME against local tumors but also often generate abscopal effect against distant metastases by activating systemic antitumor immunity (124).

To eradicate cancer cells, effector immune cells must first be activated and overcome the multiple suppressive factors in the TME. Strategies to reverse the immunosuppressive TME include the targeted inhibition of key immunomodulatory factors in the TME using inhibitors of angiogenesis (89), ICIs (60), and agents targeting immunoregulatory cells and factors (113). Off-target effect and treatment resistance greatly weaken the therapeutic effect of single treatment regimen. Therefore, a shift from monotherapy to combination therapies is essential to provide more options of available treatments. The development of novel combination therapies may help enhance the antitumor effects of current therapies and prevent the development of treatment resistance. Hydrogels provide a promising platform for the co-delivery of multiple agents targeting various components of the TME while causing minimal systemic toxicity. In addition, injectable hydrogels can also be combined with conventional treatments, such as radiotherapy and chemotherapy, to transform immunosuppressive TME to a pro-inflammatory state and amplify the antitumor immune response (50, 121, 125).

Despite the advances in injectable hydrogels, there are still several challenges that limit their clinical translation. It is necessary to determine at which stage of tumorigenesis a given treatment is most effective, and whether the effect of treatments depends on the composition of TME at the primary and metastatic sites. Although several combination systems demonstrate synergistic effects, their compositions need to be further optimized to maximize their antitumor efficacy and reduce side effects. Furthermore, future work is required to ensure that in addition to exerting antitumor effects locally and modulating the TME, hydrogels also activate systemic immune responses to prevent metastasis and tumor recurrence. Future multidisciplinary studies are warranted to design injectable hydrogel-based delivery systems for the co-delivery and sequential release of different therapeutic agents to maximize the overall therapeutic efficiency of cancer therapies and accelerate their clinical translation, especially in some late-stage cancers, such as malignant pleural effusion and malignant ascites (126).

## Author Contributions

YL and YG wrote the manuscript. JH and HJ drafted the outline for the review and revised the manuscript. BY and P-CL checked the format and content of the manuscript. All authors contributed to the article and approved the submitted version.

## Funding

This work was supported by National Natural Science Foundation of China (Grant No. 82022040 and 81874233).

## Conflict of Interest

The authors declare that the research was conducted in the absence of any commercial or financial relationships that could be construed as a potential conflict of interest.

## Publisher’s Note

All claims expressed in this article are solely those of the authors and do not necessarily represent those of their affiliated organizations, or those of the publisher, the editors and the reviewers. Any product that may be evaluated in this article, or claim that may be made by its manufacturer, is not guaranteed or endorsed by the publisher.
